# Roles dissemination and implementation scientists can play in supporting research teams

**DOI:** 10.1186/s43058-020-00107-4

**Published:** 2021-01-15

**Authors:** Rachel G. Tabak, Ana A. Bauman, Jodi Summers Holtrop

**Affiliations:** 1 Prevention Research Center in St. Louis, Washington University in St. Louis, St. Louis, MO USA; 2 Brown School of Social Work, Washington University in St. Louis, Campus Box 1196, One Brookings Drive, St. Louis, MO 63130 USA; 3 Department of Family Medicine and Dissemination and Implementation Research Program of the Adult and Child Consortium for Health Outcomes Research and Delivery Science (ACCORDS), University of Colorado School of Medicine, Aurora, CO USA

**Keywords:** Collaboration in implementation science, Funding of implementation science, Education on implementation science

## Abstract

The field of dissemination and implementation (D&I) science is rapidly growing, with many scientists seeking to apply D&I science to enhance and expand the impact of their work. As the D&I field grows and collaborations of implementation scientists with other fields flourish, a description for the roles for D&I scientists as they collaborate with researchers from other fields could be beneficial. This paper exemplifies how the D&I scientist/researcher collaborative process might work and important elements to consider in doing so, as well as provide an outline on how collaborations might progress for different project needs. This is discussed through example scenarios to consider an implementation scientists’ engagement in a research project and describe potential roles for implementation scientists in supporting research teams. We then discuss characteristics to consider when incorporating a D&I expert into a team and considerations in navigating the scenarios.

Contributions to the literature

- Contributes to the conversation as investigators find models for collaboration with the developing field of dissemination and implementation (D&I) science
- Provides potential models for collaboration in common scenarios
- Discusses characteristics for D&I experts as they are incorporated in teams, to add to the discourse on what it means to be a D&I scientist

## Background

The field of dissemination and implementation (D&I) science is rapidly growing, with many scientists seeking to apply D&I science to enhance and expand the impact of their work [1–3]. The incorporation of D&I science in scientific projects includes an emphasis among funders, encouraging plans for dissemination within a broader range of application types [4–7], and focusing on the science of D&I science. More investigators are, therefore, incorporating D&I science in their study designs both because of funding announcements, but also because they see the potential for D&I science to enhance the impact of their efforts. Several training programs and guides to foster capacity building in the field have been developed [8–20], including the development of competencies for implementation researchers [13, 21–23], but the D&I science field is nascent so widespread expertise of D&I knowledge and skills on how to collaborate with others is limited. Guidance for how to engage with D&I scientists could benefit the field. Further, as the D&I field grows and collaborations of D&I scientists with other fields flourish (e.g., infectious disease, nutrition education, cancer prevention and control, human services) [24–30], a description for the roles for D&I scientists as they collaborate with researchers from other fields could be beneficial. Investigators seeking funding to conduct studies involving D&I components need to demonstrate the research team’s capacity to successfully complete the proposed study [31] and the key role the D&I scientist plays from the beginning of a research idea/design through carrying out the research protocol and presenting the findings. Although this circumstance most often applies to projects peer-reviewed for funding consideration, the same applies to non-funded projects, to internal or community projects, and in publishing.

The goal of this paper is to discuss how the D&I scientist/researcher collaborative process might work and elements to consider in doing so, as well as provide an outline on how collaborations might progress for different project needs. We begin with an example scenario to consider a D&I scientists’ engagement in a research project and describe potential roles for D&I scientists in supporting research teams. Then, we discuss characteristics to consider when incorporating a D&I expert into a team and considerations in navigating the scenarios. These approaches are offered based on our collective experience providing consultation services that help support investigators in developing implementation science research. The authors have significant experience providing consultation on D&I science through their professional positions. AB (Co-Director) and RGT have been working with the Dissemination and Implementation Research Core (DIRC), a methods core from the Institutional Clinical and Translational Science at Washington University in St. Louis for more than 9 years. RGT is also a Co-Director of the D&I in Diabetes Core with the Center for Diabetes Translation Research at Washington University in St. Louis. JSH is the Associate Director of the D&I Program and Senior Implementation Scientist at the Adult and Child Consortium for Health Outcomes Research and Delivery Science (ACCORDS) at the University of Colorado School of Medicine and a Senior Scientific Advisor on D&I for the Agency for Healthcare Research and Quality. As consultants to our customers, we have developed toolkits and have supported several investigators receive funding for their own research through the National Institutes of Health, foundation, and internal pilot grants [32, 33].

## A decisional pathway for dissemination and implementation (D&I) research collaboration

### Working through an example scenario to describe possible pathways

We begin with examples of possible pathways based on decisions made to describe how this issue arises. To illustrate, we provide a description of how relationships between researchers might develop and evolve.

#### The actors in the scenario

Consider a D&I science expert (Dr. Implementation or Dr. I) and an investigator looking to incorporate D&I science into his/her study (Dr. Researcher or Dr. R). Dr. R is a treatment developer and/or clinical trialist with significant expertise in his/her substantive area (e.g., substance abuse treatment, HIV prevention, diabetes management interventions). Dr. R becomes interested in D&I because the inclusion of D&I science and D&I expertise into his project was specifically noted in the summary statements from his grant review. He attends a talk about D&I and sees potential in applying D&I science to his work.

#### Starting the discussion

##### Scenario 1

Dr. R happens to work at a university with a D&I research program, which provides support services. Dr. R submits a request for services through the online consultation request form, and Dr. I responds by sending Dr. R initial readings and resources to help orient Dr. R to D&I science as well as to arrange an initial meeting. At the meeting, Dr. I inquires about Dr. R’s research to help understand the research questions and how D&I science might further Dr. R’s research agenda. Dr. R also considers that D&I might be beneficial for his research question.

*Decision 1 (Incorporating D&I science into the project)*: While we recognize a study at any point along the translational continuum could benefit from a D&I perspective [34, 35], not all investigators who interact with D&I scientists and who initially express interest decide to pursue application of D&I in their work. Lane-Fall et al. lay out a mechanism for guiding this decision process, based on questions about intervention efficacy, effectiveness, and implementation [36].

Option 1: Drs. I and R decide not to collaborate at this time. This may be for many reasons. For example, they may together decide that the study is not ready for D&I (i.e., needs efficacy testing first) and Dr. R is not interested in pursuing a designing for dissemination approach to further intervention development and testing or it may be that Dr. R is not ready/able/willing to invest resources in D&I at this time. They may decide to start back at decision 1 at a later date as Dr. R moves his research agenda along.

**Fig. 1:**
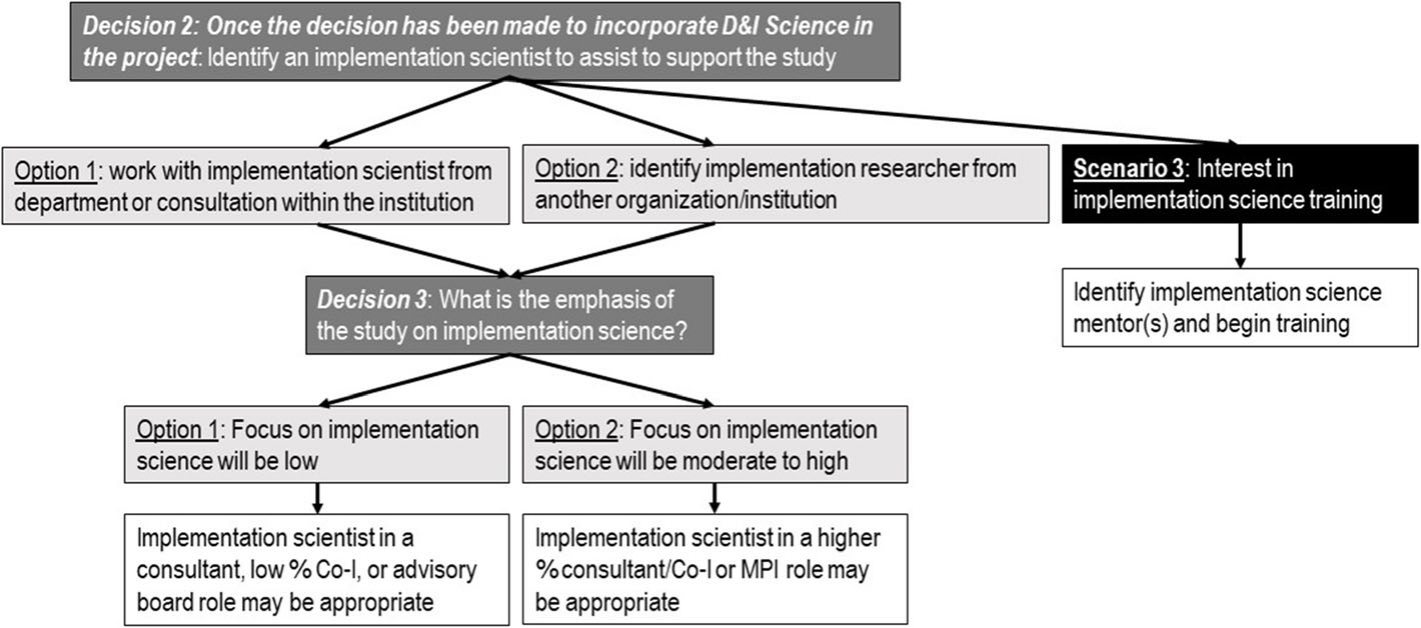
Flow of decisions once the decision has been made to incorporate D&I Science in the project

Option 2: If Dr. I and Dr. R both determine that the research question could benefit from an application of D&I science, several decisions for how the relationship could proceed are laid out in Fig. 1. This would lead Dr. R to determine the appropriate D&I investigator to support the project long-term: decision 2.

Figure 1 below outlines key decision points within in the preparation of grants, based on our experiences, and will be referred to in the discussion of decisions 2 and 3 and scenario 3.

**Fig. 2:**
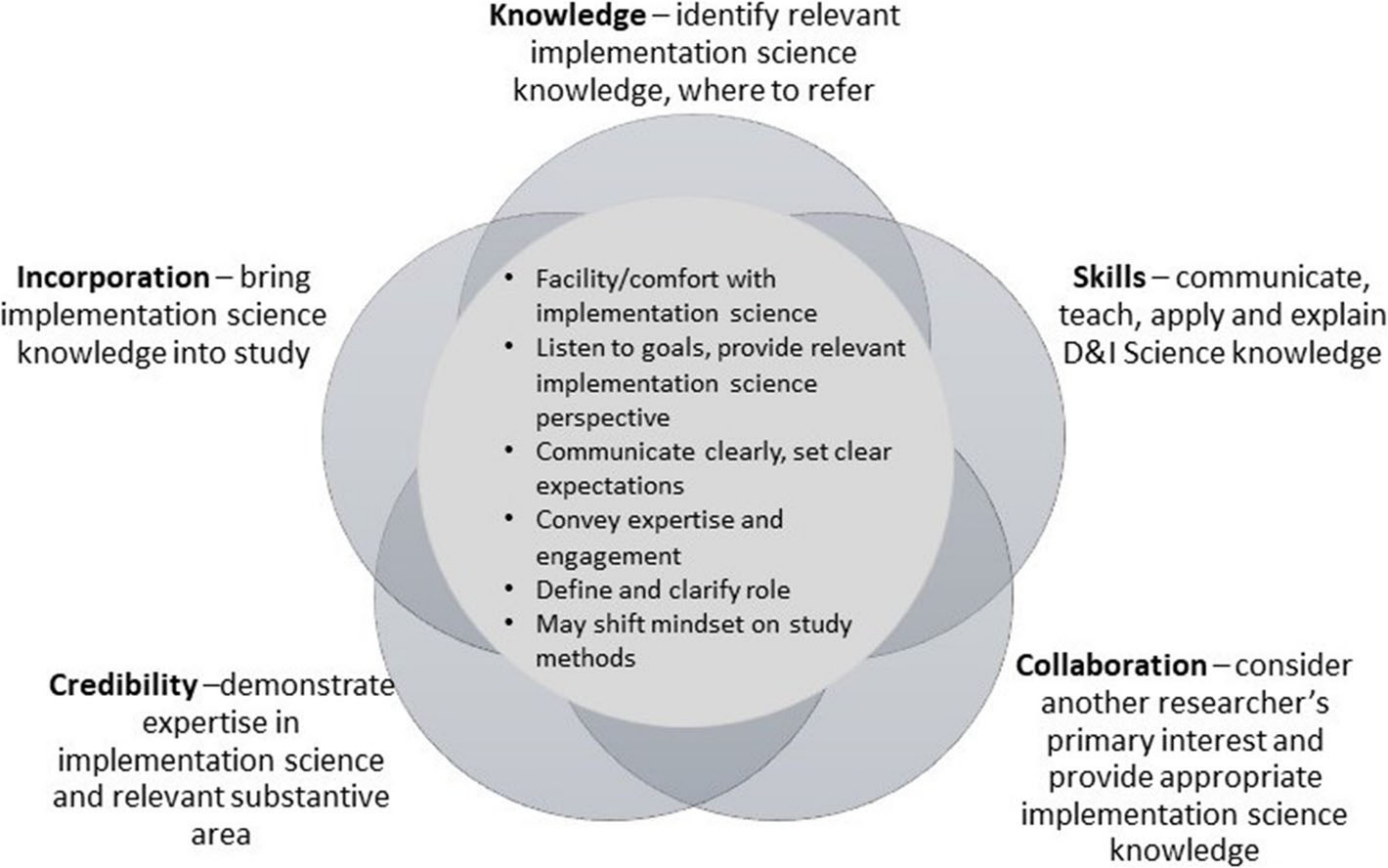
Characteristics to consider when incorporating a dissemination and implementation science expert into a team

*Decision 2 (Identifying the D&I expert)*: If Dr. R becomes interested in learning more about the D&I field, he needs to determine how best to build D&I science within his work. Although not the focus of this discussion, it is also very likely that other methods specialists specific to the D&I elements of the study join the team at this point so that these areas of expertise are represented such as economic analysis or qualitative and mixed methods expertise for example; it may be Dr. I who suggests these collaborators and makes connections. The text and Fig. 2 below describe some characteristics of a D&I scientist that Dr. R may consider in selecting with whom to partner.

Option 1: To accomplish this, Dr. I and Dr. R’s team might decide to work together, with Dr. I continuing to support R’s project. This would lead the two investigators to determine how best to work together: decision 3.

Option 2: Alternately, Dr. I might be committed to existing projects and unable to help Dr. R for the long-term or the topic of Dr. R’s study may be too far outside Dr. I’s field of interest. Dr. I could then refer Dr. R to another member of the D&I community with more aligned interest or availability if this is available. Once this D&I scientist is identified, s/he and Dr. R could move to decision 3.

*Decision 3 (Determining role of the D&I expert)*: Regardless of the D&I scientist Dr. R works with, Dr. R, working in collaboration with the D&I scientist, needs to determine what role the D&I scientist will play. Because incorporating D&I science may require changes in framing of research questions and priorities which can have impacts throughout the study design and budget, providing support in D&I science requires Dr. I to build a relationship with Dr. R. This is particularly important if D&I science is to be integrated within the study, as this requires time to understand the D&I science concepts and, importantly, to incorporate them as a way of thinking about research decisions and trade-offs. We recognize this is a critical decision in the study design process and a decision D&I scientists often help make with other subject-matter and methods experts (e.g., the strength and generalizability of the evidence base for the practice/program/policy of interest). However, we will not provide a lengthy description here, and refer readers to other literature, such as the work of Lane-Fall et al. and Curran et al. to guide this choice [36–38].

Option 1: If the study has a heavy emphasis on intervention efficacy or effectiveness (emphasis on D&I science is low), but with assessment of context or preliminary implementation strategy development, or on intervention development, but with an effort to design the intervention for dissemination, then Dr. I might have a supportive role [37, 39]. Dr. I might serve in this role as a consultant, member of an advisory board, or as a co-investigator at a small percent effort, and for example assist with ancillary measures for preliminary, contextual data collection to inform future implementation strategy development. Meanwhile, Dr. R might receive other supplementary training.

Option 2: If the study has a more central (moderate to high) emphasis on D&I, then it is important that Dr. I (or other D&I investigator) play a more prominent role as co-investigator or co-principal investigator (PI). It should be noted that often as study planning progresses, the decision of how much to embed D&I science in the study changes. For example, instead of measuring only acceptability of an intervention, now the study may also focus on understanding the components of the strategies that would facilitate adoption and sustainability of the intervention. When this is the case, Drs. I and R may consider increasing the role of Dr. I to a higher percent effort consultant or co-investigator or to a co-PI.

In both options 1 and 2, Dr. I plays a central role in the research providing key contributions to the project and has a specific number of hours to provide support on the study both pre- and post-award. As part of option 1, Dr. I could also serve as a member of an Advisory Board. In this case, her involvement in the study is defined by encounters with the research team, who would present about the progress of the study and consult on specific topics [40].

##### Scenario 2

Dr. R happens to work in the field or at a university where there is no formal D&I program. Dr. R is still interested in incorporating D&I science in his study, so, in this case, Dr. R could search to determine if there is someone with D&I expertise at his own institution by asking colleagues or searching PubMed or journals commonly accessed for D&I articles as identified by Norton et al. [41]. Also, there may be a D&I scientist at another institution with a content-area interest that may be willing to be a co-investigator or paid consultant. Finding a D&I scientist who is available to collaborate can be challenging due to lack of capacity in the D&I field overall and is discussed below under the section called “Considerations in Navigating the Scenarios.” Once a D&I scientist is identified, this scenario can follow from decision 1 as in scenario 1 above. However, in either case, the hypothetical Dr. R might decide to pursue his own D&I training.

##### Scenario 3

As noted above, an alternative path could be if Dr. R becomes interested in D&I science at a deeper level himself or is unable to find a D&I collaborator and decides to pursue some level of D&I training (right side of Fig. 1). The path of training can include a combination of formal training as well as informal mentorship, apprenticeship style training, and mentored work applying D&I science in the field. There are many formal and informal training programs emerging for this researcher to investigate and he could consider training grants with a focus on D&I research. In this case, Dr. R would benefit greatly from a D&I scientist such as Dr. I as a mentor. The many resources to support D&I training and education and for identifying D&I collaborators have been reviewed and collected in several compilations [14]. We do not aim to replicate these resources; rather, we point readers to resources such as websites [42–48] and trainings [49–55] they may look to as they explore the decisions discussed [14, 56].

### Summarizing potential roles for a D&I scientist

Based on the scenarios and decisions described above, Table 1 summarizes the types of roles for researchers trained in D&I science when collaborating on research studies with researchers from other fields.

**Table 1:** Potential roles for a dissemination and implementation (D&I) scientist as a collaborator

Example	Emphasis on D&I	D&I scientist (Dr. I)^**a**^ role	D&I scientist (Dr. I)^**a**^ engagement	Content area expert (Dr. R)^**b**^ role
Exploratory aim to assess context or implementation outcomes (e.g., acceptability, feasibility, cost)	Low	● Understand context ● Assess implementation outcomes ● Incorporate designing for dissemination principles	● Consultant ● Advisory board ● Co-investigator	PI
Preliminary aim or preliminary study to assess context, dissemination channels, or feasibility	Low	● Understand context ● Assess implementation outcomes ● Inform dissemination and/or implementation strategy	● Consultant ● Advisory board ● Co-investigator	PI
Preliminary or pilot study to assess context, dissemination channels, or feasibility	Low/moderate	● Understand context ● Assess implementation outcomes ● Pilot dissemination and/or implementation strategy	● Consultant ● Advisory board ● Co-investigator	PI
Secondary aim is D&I science specific	Moderate/high	● Understand context ● Assess implementation outcomes ● Develop, tailor, and/or test implementation strategies	● Co-investigator ● Multiple PI	● PI ● Multiple PI
Primary aim is D&I science specific	High	● Understand context ● Assess implementation outcomes ● Develop, tailor, and/or test dissemination and/or implementation strategies	● Multiple PI	● PI ● Multiple PI

^a^Researcher with D&I expertise

^b^Researcher with subject-matter expertise and not D&I science expertise

## Characteristics to consider when incorporating a D&I expert into a team

Beyond the decision points regarding the extent to which D&I is appropriate in a study and a D&I expert is needed, there is more to building a successful collaboration. Other fields have provided descriptions of how scientists may collaborate in multi-disciplinary teams (e.g., statisticians [57–63]) and characterized the tasks they complete in consultation meetings (e.g., learning content area of collaborator, reviewing protocol, etc.) [64]. Gilliland et al. [65] described seven characteristics of a translational scientist: boundary crosser, domain expert, team player, process innovator, skilled communicator, systems thinker, and rigorous researcher. We build on this work to describe possible pathways of collaborative work between D&I researchers and experts from other fields. D&I scientists may be particularly well suited to this type of multi-disciplinary collaboration given many D&I scientists are also experts in another type of research and bring diverse substantive backgrounds and training experience (e.g., mental health, infectious disease, public health).

In Fig. 2, we present a model for considering characteristics important specifically for the D&I consultation relationship. The model is presented to resemble a flower with a big core of overlapping areas, but with distinct pedals to signify important elements. Early-stage investigators looking to develop expertise in D&I science for a variety of roles, and to expand the capacity of the field, may consider these characteristics in addition to the competencies described elsewhere [13, 21–23] for developing D&I expertise to apply in their own work.

### Knowledge

An important element of a successful collaboration is that the D&I scientist has knowledge of D&I science methods to bring to the relationship. The hypothetical Dr. I should have a solid basis of understanding of D&I frameworks and theories, implementation strategies and methods, methods to understand context, adaptation, and expected outcomes. Sometimes, specific expertise and experience with a particular model or framework and/or implementation strategy is needed and extra consultation may be required [13, 21]. Knowledge of D&I can be demonstrated in how the D&I frameworks are aligned with the methods, designs, and measures of the project, as well as with contextual determinants (e.g., barriers and facilitators to implementation) and the plans for developing and tailoring implementation strategies [31, 66, 67].

### Incorporation

This is the ability to bring the knowledge of D&I and apply it to the situation at hand. This means taking D&I knowledge and being able to use it in crafting new research and understanding outcomes from existing findings. Incorporating D&I science in a grant or study goes beyond applying the knowledge within the D&I scientist’s own area of research; it involves applying D&I science to another investigator’s line of research. As D&I scientists in numerous roles, we have collaborated with experts in a range of disciplines, such as cancer and sickle cell disease, and supported D&I science in these diverse disciplines. We have found that part of what we do is “translate” D&I in these different disciplines for other researchers, which goes beyond just knowing the D&I science.

### Skills

A wide variety of skills are important here, similar to the characteristic identified by Gilliland et al. [65] of being a “skilled communicator.” Skills can be both broad (i.e., the skill of working collaboratively and communicating appropriately) or specific to D&I (i.e., the skill of selling the importance of D&I to different investigators, of framing a D&I proposal for different study sections or the skill to communicate, teach, and explain D&I science knowledge). The ability to communicate about D&I science to diverse audiences contributes to a project’s development, execution, and impact by supporting engagement of stakeholders with the D&I science aspect of the work.

### Credibility

The D&I scientist needs to be considered as having substantive knowledge in D&I science to be credible to reviewers and to support a D&I project. Sometimes, the D&I scientist needs to be senior or highly networked, especially if the proposed work is a large competitive grant or program, and knowing and tapping into a network of other D&I experts for insight is necessary.

### Collaboration

The D&I scientist should be a person who is able to consider another researcher’s primary interest and support that work. This goes beyond being a boundary crosser (i.e., “Breaks down disciplinary silos and collaborates with others across research areas and professions to collectively advance the development of a medical intervention”), to work across disciplines [65], but involves prioritizing another investigator’s research agenda. It is important to inform and point out places of challenge, but not take over the agenda.

Taken together, these relationships often require for the hypothetical Dr. I as the D&I scientist to have D&I science knowledge and skills in the abstract, and the ability to apply them to an area of research that is not necessarily her primary discipline. Once a person is comfortable with the science, one can collaborate with those new to D&I science (such as Dr. R and his team) and support them in understanding D&I science concepts and the value of their application. This allows investigators such as Dr. R to truly integrate the science within his study and research agenda. For example, Dr. R may ask Dr. I to assist in selecting and applying a theory, model, or framework. In-depth knowledge of a wide range of theories, models, and frameworks facilitates advising Dr. R. However, it is also necessary for Dr. I to understand Dr. R’s goals and to support him in utilizing the selected framework in the proposed work. Similarly, Dr. I may assist Dr. R in crafting a D&I science question/component for studies at different phases. These require deep knowledge of D&I science, facility and flexibility with the evidence base, awareness of the many resources available (e.g., books, literature, web resources), and skills in research design.

The competitive nature of grant funding requires Drs. R and I to clarify their relationship, convey how Dr. I is engaged with the project team, and articulate how Dr. I’s expertise contributes to the study. For example, Dr. I’s biosketch should reflect publications in D&I broadly and, if possible, in the components of D&I science specific to the proposed work (e.g., assessing implementation context, building and testing implementation strategies). If the D&I scientist has conducted work in the relevant substantive area, those are publications which could be highlighted. While it may not be possible for all collaborations, pilot work related to the D&I science research question, which could be co-produced, is also beneficial. Regardless of the relationship Drs. I and R decide upon, clear description of the role Dr. I will play in Dr. R’s study team is important, for example, statements in a grant proposal text and/or budget justification such as “Dr. I is the D&I science lead and will contribute to the study through XX activities” (Proctor et al. [31]). The resources available for D&I should also be outlined in the facilities and resources’ sections specifying resources beyond the individual D&I expert. Further, for any role, it is critical for Dr. I to be engaged enough in the study and proposal development so that the D&I science components are clear and well-integrated. It likely shows through in the text of a grant proposal and/or manuscript when a D&I scientist is merely named on the project but does not play a central role in that team. As noted above, the level of necessary engagement depends on the study aims and how integral D&I science is to the project, but adequate ongoing engagement is nonetheless important to clearly describe how D&I methods strengthen the study.

Finally, Dr. R and Dr. I need to work together effectively and follow general ground rules of effective collaboration. This includes having the bandwidth to be an effective collaborator with timely review and follow-up on assigned tasks, clear and honest communication, and being respectful and considerate of each other. This may be especially important for investigators coming from different points of view to start, for example, perspective on the importance of randomized controlled trial study designs. Discussion and patience are needed to come to a common understanding of the choices to achieve the main objectives of the study. Time preparing a proposal, paper, or other collaborative product is useful to determine if these relationships can be productive and professional. Specific strategies such as collaboratively completing a PRECIS-2 “wheel” or navigating resources for selecting theories, models, and frameworks early on can help the hypothetical Dr. R weigh the implications of a more pragmatic approach and the importance of implementation context and outcomes to the research process. Also, the use of other tools and resources [32, 56] can help identify potential “pain points” in perspectives and work through the process of clarifying the research needs.

Overall, there are many decision points in study development in general and to navigating the role of a D&I scientist specifically. It is of course important to consider logistical and budget implications of these decisions, the types of grants being prepared, and the evidence of the intervention being studied. These have implications for the type of role Dr. I, as a D&I scientist, has in the study.

## Considerations in navigating the scenarios

These scenarios are presented as relatively smooth, linear processes of developing research collaborations and acquiring training (formal and/or mentored). However, like any transdisciplinary work, there may be challenges in identifying D&I experts with availability. For example, many universities do not have D&I programs, so requesting a free consult through an organized request system with a D&I scientist is not always an available option. Therefore, requesting consultation from an individual investigator who is not part of a D&I program to provide consultation might be challenging [14, 41]. Even at universities with D&I programs, it is possible all the D&I experts are fully funded and do not have time available for years.

For investigators seeking training, increasingly, training programs for researchers to learn how to conduct D&I are available; however, there is no consensus on what constitutes sufficient training to conduct work independently without assistance [56, 68–70]. Further, while knowledge of D&I may be useful, it may not be perceived as contributing to a researcher’s being “known” as a D&I scientist on a competitive grant proposal. With regard to mentored or apprentice-style training, an additional problem with capacity is that K and other career awards do not provide funded time for the mentor, as well as similar challenges matching with a mentor with availability and relevant expertise. To increase capacity of a senior/junior partnership where two D&I scientists are included on a proposal, with the senior at a smaller percent of effort and the junior at a larger percent effort with the senior advising and overseeing the junior, can be considered. This has many advantages, as a capacity building experience for the junior investigator; however, this leaves the senior on a lot of projects at a small percent effort, which is challenging. This highlights the difficulty of spreading over numerous projects from diverse substantive areas, which is not always valued in promotion and tenure decisions, and which may discourage D&I scientist such as Dr. I from joining collaborations. This is a particular problem for a field that values transdisciplinary work. These issues are not dissimilar from other fields, such as in economics and statistics.

Other issues relate to those typical to many collaborations where the hypothetical Dr. R essentially does not provide an appropriate set-up for collaboration. Some examples include the following: Dr. R approaches Dr. I very close to a grant deadline, which does not allow time for discussion and consideration of the alternative designs or methods that are more common in D&I research, or Dr. R has an efficacy intervention that is not likely to work in the “real world” and is unwilling to modify it for D&I potential. There is no one answer to these challenges; however, they do exist and are worth acknowledging. In these instances, the hypothetical Dr. I needs to have one of those “conscious conversations” with Dr. R to resolve these differences or collaboration may not proceed.

## Discussion

Because D&I science is emerging rapidly, there is a growing need for these experts to support investigators new to the science. Incorporating a trained and collaborative D&I scientist has the potential to improve funding and publishing success for a study with a D&I emphasis or components. Providing quality efforts and services in a timely manner can help the D&I science field continue to grow.

The options and scenarios described above are not designed to be exhaustive, but to stimulate discussion and contribute to a structure. There are likely many ways the dynamic between Drs. I and R might evolve, and the relationship and roles are often iterative and change over time. Our goal has been to further the discussion about the needs of the field of D&I research and make it as impactful as possible. This is based on our experiences and those of other D&I programs gathered through collaborative input. We believe this information will generalize to D&I scientists in many programs. Future research could examine the impacts of levels of engagement or training in D&I on successful funding of grant applications or acceptance of publications and especially on clinically relevant implementation and health outcomes. Future investigation could also explore the use of D&I scientists in consultant roles in research over time to get a sense of trends in this area as well as opportunities to support early- and mid-career D&I scientists including building experience with consultative roles (for example through embedded research support or lighter consultation).

## Conclusion

Building teams, which incorporate implementation science, can follow many paths. Given the ongoing development of implementation science as a field, the conversation of what makes an investigator a D&I scientist and how they can best partner with investigators seeking these skills is evolving, but several key roles and criteria can be identified.

## Data Availability

Not applicable

## Abbreviations

D&I: Dissemination and implementation
PI: Principal investigator
